# Leukocyte- and platelet-rich fibrin in endoscopic endonasal skull base reconstruction: study protocol for a multicenter prospective, parallel-group, single-blinded randomized controlled non-inferiority trial

**DOI:** 10.1186/s13063-023-07492-w

**Published:** 2023-07-31

**Authors:** Birgit Coucke, Anaïs Van Hoylandt, Mark Jorissen, Jeroen Meulemans, Thomas Decramer, Johannes van Loon, Vincent Vander Poorten, Tom Theys, Laura Van Gerven

**Affiliations:** 1 Research Group Experimental Neurosurgery and Neuroanatomy and Leuven Brain Institute, Department of Neurosciences, KU Leuven, Belgium; 2 Allergy and Clinical Immunology Research Group, Department of Microbiology, Immunology & Transplantation, KU Leuven, Leuven, Belgium; 3 Neurosurgery, University Hospitals Leuven, Leuven, Belgium; 4 Otorhinolaryngology, Head and Neck Surgery, University Hospitals Leuven, Leuven, Belgium; 5 Laboratory of Experimental Otorhinolaryngology, Department of Neurosciences, KU Leuven, Leuven, Belgium; 6 Department of Oncology, Section Head and Neck Oncology, KU Leuven, Leuven, Belgium

**Keywords:** Cerebrospinal fluid leakage, Endoscopic endonasal transsphenoidal approach, Dura, Regenerative medicine, Skull base, Prevention, Leukocyte- and platelet-rich fibrin

## Abstract

**Background:**

Recent advances in endoscopic endonasal transsphenoidal approaches (EETA) for skull base lesions have resulted in a significant increase in extent and complexity of skull base defects, demanding more elaborate and novel reconstruction techniques to prevent cerebrospinal fluid (CSF) leakage and to improve healing. Currently, commercially available fibrin sealants are often used to reinforce the skull base reconstruction. However, problems have been reported regarding hypersensitivity reactions, efficacy, and costs. This trial aims to investigate autologous leukocyte- and platelet-rich fibrin (L-PRF) membranes as an alternative for commercially available fibrin glues in EETA-related skull base reconstruction reinforcement.

**Methods/design:**

This multicenter, prospective randomized controlled trial aims to demonstrate non-inferiority of L-PRF membranes compared to commercially available fibrin sealants in EETA cases (1) without intra-operative CSF-leak as dural or sellar floor closure reinforcement and (2) in EETA cases with intra-operative CSF-leak (or very large defects) in which a classic multilayer reconstruction has been made, as an additional sealing. The trial includes patients undergoing EETA in three different centers in Belgium. Patients are randomized in a 1:1 fashion comparing L-PRF with commercially available fibrin sealants. The primary endpoint is postoperative CSF leakage. Secondary endpoints are identification of risk factors for reconstruction failure, assessment of rhinological symptoms, and interference with postoperative imaging. Additionally, a cost-effectiveness analysis is performed.

**Discussion:**

With this trial, we will evaluate the safety and efficacy of L-PRF compared to commercially available fibrin sealants.

**Trial registration:**

ClinicalTrials.gov NCT03910374. Registered on 10 April 2019.

**Supplementary Information:**

The online version contains supplementary material available at 10.1186/s13063-023-07492-w.

## Administrative information

We used the Standard Protocol Items: Recommendations for Interventional Trials (SPIRIT 2013) checklist when writing our report [1]. The numbers in curly brackets in this protocol correspond to the SPIRIT checklist item numbers.

**Table:** 

Title {1}	Leukocyte- and platelet-rich fibrin endoscopic endonasal skull base reconstruction: a multicenter prospective, randomized controlled trial.
Trial registration {2}	ClinicalTrials.gov (ID: NCT03910374) https://clinicaltrials.gov/ct2/show/NCT03910374 KU/UZ Leuven: S61636
Protocol version {3}	V5 dd 9-11-2021
Funding {4}	FWO TBM grant T003018N The design of the study has been reviewed by FWO, but FWO has no role in data collection, analysis, and interpretation of data nor in writing the manuscript.
Author details {5a}	Otorhinolaryngology-Head and Neck Surgery, UZ Leuven, Leuven, Belgium Neurosurgery, UZ Leuven, Leuven, Belgium
Name and contact information for the trial sponsor {5b}	UZ Leuven Herestraat 49, 3000 Leuven, Belgium +32 16 33 22 11
Role of sponsor {5c}	The sponsor has no role in the design of the study, study conduct, collection of data, analysis nor in writing of trial manuscripts.

## Date and version identifier {3}

**Table:** 

**Date**	**Version**
16-5-2018	Original (v1)
30-8-2018	Amendment no.1: Sample size recalculation (v3)
5-2-2020	Amendment no.2: Addition of ICF translated in English
17-3-2020	Digital follow-up possible (COVID-19 restrictions)
8-5-2020	Amendment no. 3: Addition of ICF translated in French
30-3-2021	Amendment no. 4: Addition of quality control measures (v4)
30-6-2022	Amendment no. 5: Addition of a third participating center: UZ Gent (v5)

## Background and rationale {6a}

The endoscopic endonasal transsphenoidal approach (EETA) to the skull base is a minimally invasive surgical technique that is used to treat a wide range of conditions affecting the skull base, such as pituitary tumors, suprasellar tumors and other diseases of the skull base. EETA is performed through the nasal cavity, without the need for an open craniotomy or facial incision, which leads to a faster recovery, reduced postoperative pain, and a lower risk of complications. However, the nasal cavity is a delicate and complex area that requires precise surgical techniques and can be susceptible to bleeding and inflammation. Additionally, recent advancements in endoscopic endonasal approaches (EEA) for skull base lesions have resulted in a significant increase in the extent and complexity of skull base defects, demanding more elaborate and novel reconstruction techniques to improve healing and prevent reconstruction failure. One major complication of EETA is postoperative cerebrospinal fluid (CSF) leakage. Our recently published systematic review, summarizing data from 113 studies, showed an average postoperative CSF leakage rate of 4.1% in endoscopic transsphenoidal surgeries [2]. A well-known risk factor for postoperative CSF leak is the presence of intraoperative leakage, in case of malignancy or, in cases where a thinned arachnoid herniates into the sella. As the pituitary is located in the diaphragma sellae, a double fold of the dura, large tumors may extend through the central opening of the diaphragm, resulting in a typical dumbbell shape, with an increased risk of CSF leak.

Postoperative leaks may require revision surgeries and/or external CSF drainage. Adequate dural closure is necessary to prevent CSF leak-related complications such as infections and intracranial hypotension. Currently, commercially available fibrin sealants, including Tisseel® and Tachosil®, are considered the gold standard for reinforcement of dural closure, due to their hemostatic, adhesive, and sealant properties [3]. However, problems regarding efficacy, cost, and safety have been reported [4, 5]. As such, commercially available fibrin sealants are expensive and may risk inducing hypersensitivity reactions.

**Fig. 1:**
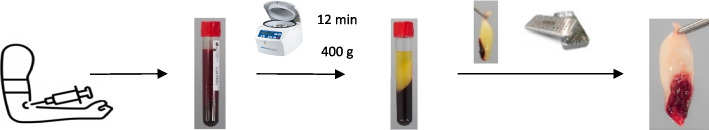
Leukocyte- and platelet-rich fibrin (L-PRF) preparation protocol. Membrane preparation after blood collection in glass tubes and centrifugation for 12 min (400 g), compression of the fibrin clot

Leukocyte- and platelet-rich fibrin (L-PRF) is a biologically active material that is derived from the patient’s own blood [6]. L-PRF can be prepared in solid form as membranes or as plugs, or as a liquid. The main advantage of L-PRF is that it is completely autologous, thereby minimizing the risk of immunological reactions. Additionally, L-PRF is non-invasive, inexpensive, and can be prepared in a 20-min time span. Solid L-PRF is obtained by centrifuging a small sample of blood for 12 min at 400 g in glass or silica-coated tubes (Fig. 1), during which the coagulation cascade is initiated, and different blood components are separated. The resulting blood clot is the actual L-PRF, situated right above the red blood cell layer. It is in fact a fibrin matrix that contains a high concentration of platelets, leukocytes, and growth factors. These growth factors would promote tissue healing and reduce inflammation, which makes L-PRF an attractive option to use in a variety of surgical procedures. Analogously, liquid L-PRF glue can be prepared in plastic-coated tubes. Because of the hydrophobic surface and shorter centrifugation time (3 min), the product maintains its native fibrinogen and only coagulates in contact with other tissues [7].

Recently, platelet concentrates including L-PRF are of increasing interest in EETA to enhance surgical outcomes, improve tissue healing and minimize postoperative bleeding. Regarding postoperative CSF leakage, some preliminary retrospective clinical studies showed comparable results of L-PRF with commercially available fibrin sealants [8–10]. The fibrin matrix of L-PRF has been shown to provide a scaffold for new tissue growth which, in combination with the local release of growth factors from leukocytes and platelets, would promote angiogenesis resulting in improved healing [11, 12].

The preparation of L-PRF is a relatively simple process that can be performed in the operating room in parallel with the surgery. Solid L-PRF membranes in EETA can be applied in different ways, depending on the size and complexity of the lesion. For example, L-PRF with a nasoseptal flap, abdominal fat grafts, or (allogenic or autologous) fascia lata are possible combinations. The application of liquid L-PRF is less interesting in EETA, because of the vertical surgical plane and required coagulation time. The indications for the use of L-PRF are still being defined and need further investigation. In this study, we aim to investigate L-PRF membranes as an alternative to commercially available fibrin sealants for dural closure after EETA.

## Objectives {7}

The general aim of this study is to evaluate the potential of L-PRF in dural closure reinforcement in endoscopic endonasal skull base reconstruction. We aim to show non-inferiority of L-PRF compared to the current standard practice. In addition, we want to demonstrate that L-PRF is more cost-effective than standard commercially available fibrin sealants. Risk factors and possible complications will be evaluated to validate safety.

### Trial design {8}

This study is a multicenter, single-blinded, prospective randomized controlled trial with subjects randomized 1:1 in two parallel groups, i.e., experimental (L-PRF treatment) and active control (commercially available fibrin sealants).

In this study, the general objective is to show non-inferiority of L-PRF as a closure technique compared to commercially available fibrin sealants in endoscopic transsphenoidal surgery. This prospective randomized controlled trial was approved by the local Ethical Committee (UZ Leuven (S61636), AZ Sint-Jan Brugge, UZ Gent) and is registered at ClinicalTrials.gov (ID: NCT003910374). Recruitment started on 07 November 2018.

## Methods: participants, interventions, and outcomes

### Study setting {9}

This study is recruiting patients admitted to the Department of Neurosurgery of the University Hospitals Leuven (UZ Leuven), AZ Sint-Jan Brugge, and UZ Gent for endoscopic endonasal transsphenoidal procedures.

### Eligibility criteria {10}

Before initiation of any study procedures, participants (or their legal representative) must provide written informed consent (Additional file 2). Inclusion criteria are patients with lesions in the sellar or parasellar region with a minimum age of 18 years (Table 1). As this study includes a strict follow-up scheme with an additional visit, patients can only enroll if they agree to adhere to the follow-up schedule.

**Table 1:** Eligibility criteria

Inclusion criteria	Exclusion criteria
Age ˃18 years	Age ˂18 years
Lesions of the sellar/parasellar region	Any underlying rhinological condition which may interfere with the obtained results
Informed consent signed	Participation to other clinical studies with drugs or medical devices
Willingness to adhere to visit schedule	

Patients with any underlying rhinological condition that may interfere with the obtained results, for example, nasal polyps, cannot be included. Participation to other clinical trials with study drugs or devices is another exclusion criterion.

### Who will take informed consent? {26a}

The neurosurgeon will inform the patients about the study at the time of planning the elective surgery. The study coordinator sends the information brochure beforehand. Trained study staff obtains the informed consent before the surgery, i.e., at the day of hospital admission which is usually 1 day before the surgery. Specialized consent forms are available for interpreters and — in case the patient is not capable to give consent — for legally authorized representatives. In such cases, it is the physician who determines the individual’s capacity to give consent.

### Additional consent provisions for collection and use of participant data and biological specimens {26b}

There are no plans for additional studies using the data collected in this trial. No biological specimens are collected.

## Interventions

### Explanation for the choice of comparators {6b}

At present, commercially available fibrin sealants are considered the gold standard for dural closure reinforcement after EETA. Tisseel® (Baxter, Deerfield, IL, USA) and TachoSil® (Corza, Düsseldorf, Germany) are used as standard of care in endoscopic transsphenoidal skull base reconstruction at UZ Leuven, UZ Gent, and AZ Sint-Jan Brugge. TachoSil® is approved by the European Medicines Agency (EMA) and Food and Drug Administration (FDA) for this indication.

### Intervention description {11a}

A team of neurosurgeons and otorhinolaryngologists perform the surgery according to local standards. If a patient is assigned to be treated with L-PRF, the use of commercially available fibrin sealants for sellar closure is not allowed. However, if deemed necessary by the surgeon, additional autologous (pericranium, muscle, fat grafts…) or allogenic (fascia lata) and hemostatic materials (cellulose sponges such as Spongostan® (Ethicon Biosurgery, Johnson and Johnson, New Brunswick, NJ, USA), Floseal® (Baxter, Deerfield, IL, USA), or Surgicel® (Ethicon Biosurgery, Johnson and Johnson, New Brunswick, NJ, USA)), can be used in both groups.

The L-PRF membranes are prepared during surgery. In order to ensure protocol adherence, the L-PRF preparation is performed by the study coordinator for study subjects assigned to treatment with L-PRF. In order to guarantee an optimal membrane formation, timing is of particular importance in this procedure. Therefore, the arterial line is flushed and immediately 20 mL of arterial blood is collected in a sterile syringe, transferred to two sterile 10-mL glass tubes (A-PRF®, Process for PRF, Nice, France), and instantaneously put in a centrifuge at 2700 rpm (400 g) (IntraSpin®, Intra-Lock, Boca Raton, FL, USA). During the transfer to the glass tubes and the start of the centrifugation, a second batch of 20 mL arterial blood is collected in a sterile syringe. This batch is transferred to 10-mL glass tubes as well, while the first centrifugation process is interrupted, and for ensuring a precise timing, the tubes are added into the centrifuge when decelerated. Then the centrifuge is instantly restarted at 2700 rpm for 12 min. If deemed necessary, an additional 20-mL syringe can be collected to fill two more 10-mL glass tubes and to be added to the centrifuge in a similar way.

After the centrifugation process, the presence of a dense clot right above the red blood cell layer in the tubes is verified. If these are not present, the tubes are put aside for 10 min and inspected again. The tubes are opened and presented to the scrub nurse for removing the clot with sterile forceps. The clot is placed in an Xpression Box® (Intra-Lock, Boca Raton, FL, USA) and gently compressed by placing the compression plate and the lid on top. After 5 min, the membranes are ready for surgical application by placing them on the dural defect. If necessary, the membranes can remain in the Xpression box® for up to 3 h until application. An instruction video of the L-PRF preparation process is available at http://doi.org/10.5281/ZENODO.8095217 [13].

### Criteria for discontinuing or modifying interventions {11b}

After allocation to the experimental or control arm, several circumstances—specified as perioperative exclusion criteria- can still lead to excluding the patient from the study. A patient allocated to the control arm may be excluded in case of a clear indication for a hypersensitivity reaction to commercially available fibrin sealants. Patients allocated to L-PRF treatment may be excluded if the L-PRF preparation process fails or if the product is considered of insufficient quality. In case the surgeon identifies other intraoperative findings that may constrain the conduct of the study procedure the subject can be excluded as well, to ensure optimal patient care in that situation. For example, when the damage to the dura is exceptional, the surgeon may opt for a multilayer reconstruction using multiple grafts, commercially available sealants, and autologous materials. Furthermore, study participants can withdraw from the study at any moment, implying that all subsequent study procedures and data collection are discontinued.

### Strategies to improve adherence to interventions {11c}

No strategies for intervention adherence are necessary, as the intervention is administered at a single time point during surgery.

### Relevant concomitant care permitted or prohibited during the trial {11d}

All other forms of treatment are permitted; however, patients are not allowed to participate in other clinical studies with investigational drugs or devices.

### Provisions for post-trial care {30}

In accordance with the Belgian Law relating to experiments on human persons (May 7, 2004) the Sponsor shall assume, even without fault, the responsibility of any damages incurred by a study patient and linked directly or indirectly to the participation to the study and shall provide compensation therefore through its insurance.

### Outcomes {12}

The primary end point is the success rate of both techniques at 3 months postoperative, which means the absence of CSF leak that needs surgical revision or any other intervention e.g., lumbar drainage, repeat imaging, or longer hospitalization. Success rate in both groups will be reported in proportions with a 95% confidence interval.

Secondary endpoints include identification of potential risk factors for reconstruction failure, assessment of potential interference of reconstruction material with post-operative imaging, analysis of the effect of the treatment on quality of life (QoL), and post-operative rhinological symptoms. Rhinological symptoms are assessed based on specific validated questionnaires, i.e. visual analog scale (VAS) (for runny nose, nasal itches, sneezing, nasal congestion, facial pain, headache, loss of smell), Sino-Nasal Outcome Test-22 (SNOT-22) and Skull Base Inventory (SBI).

The outcomes will be assessed at six study visits within a time frame of 1 year (Table 2), and a comparison will be made between the two groups considering the change to baseline (preoperative visit). Data will be presented as mean and standard deviation or median and interquartile range for continuous variables (including VAS score, SBI score, SNOT-22 score) or as proportions with 95% confidence intervals for categorical variables (intraoperative leak, complications, surgical indication, or type of tumor).

**Table 2:** Outcome assessment

**Procedure**	**Visit 1** *Screening*	**Visit 2** *Allocation*	**Visit 3** *FU1*^a^	**Visit 4** *FU 2*^b^	**Visit 5** *FU 3*^c^	**Visit 6** *FU 4*^d^
**Enrolment**						
Eligibility screening	X					
Informed consent	X					
Randomization		X				
**Interventions**						
Endoscopic endonasal transsphenoidal surgery		X				
Sellar floor reconstruction/closure		X				
**Assessments**						
Medical history	X					
EQ-5D	X		X	X	X	X
VAS	X		X	X	X	X
SNOT-22 questionnaire	X		X	X	X	X
SBI	X		X	X	X	X
MR imaging	X				X	X
Cost/material		X				
Intraoperative CSF leakage		X				
Nasal endoscopy to assess CSF leakage			X	X	X	X
Nasal endoscopy to assess wound healing			X	X	X	X
Nasal endoscopy to assess infection			X	X	X	X
Assessment of adverse events		X	X	X	X	X

*CSF *cerebrospinal fluid, *EQ-5D *EuroQol-5D, *SBI *Skull Base Inventory, *SNOT-22* Sino-Nasal Outcome Test with 22 items, *FU *follow-up, *VAS *visual analog scale

^a^2 weeks postoperative

^b^4 weeks postoperative

^c^3 months postoperative

^d^1 year postoperative

For the cost-effectiveness analysis, the “healthcare payer” perspective will be applied. This means that all direct treatment-related costs as well as costs related to the follow-up will be included in the analysis. In the cost analysis, the following elements will be considered: used materials during surgery (units), surgery duration (minutes), intensive care hospitalization time (days/hours), duration of hospitalization (days), associated facility and staff resources. These data will mainly be retrieved from the hospital records. The most recent unit prices at the time of analysis will be applied. Depending on the primary outcome, i.e., the effectiveness of sellar reconstruction, and the clinical effects of both treatments, either a cost-utility or a cost minimization analysis will be performed [10, 11]. In addition, the use of EQ-5D-3L to calculate QALYs at 6–12 weeks postoperative will be explored to compare EQ-5D utility scores to other outcomes described above.

### Participant timeline {13}

**Fig. 2:**
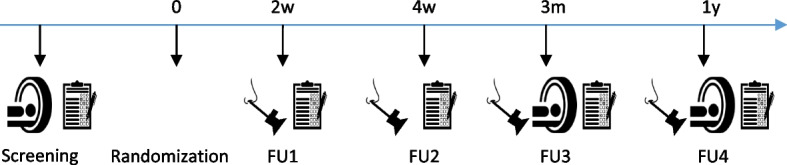
Flowchart of “L-PRF in endoscopic endonasal skull base reconstruction.” After obtaining informed consent, patients are screened and asked to complete general and disease-specific quality of life (QoL) questionnaires (EQ-5D, VAS, SNOT-22, and SBI). Preoperative MR images are assessed for tumor size and location. During surgery, study subjects are randomized into experimental arm (treated with L-PRF) and control arm (commercially available fibrin sealants). Clinical follow-up visits with an endoscopic examination of the operation wound are organized at 2, 4, and 12 weeks postop. At these visits, QoL questionnaires are completed as well. MR imaging is performed at 12 weeks postop

The participant timeline is presented in Fig. 2.

#### Screening

At the day of hospital admission (usually 1 day before surgery), informed consent is obtained and eligibility criteria are checked. A medical history questionnaire with identification of potential risk factors for reconstruction failure and post-operative CSF leaks will be filled out. The patient will be asked to complete nasal symptom scoring (SNOT-22, SBI, VAS scoring) and EQ-5D-3L questionnaires to assess the quality of life. Pre-operative MR images will be evaluated.

#### Randomization

The EETA will be performed according to local standards. During surgery, patients are randomized into experimental (L-PRF) and control (commercially available fibrin sealant) groups. Defects after closure and the need for muscle or fascia grafts are recorded. Visible perioperative CSF leakage and other complications are assessed. For each surgery, all used materials are registered and exact costs will be calculated.

#### Outpatient follow-up

The patient is seen at the outpatient clinic at four occasions to evaluate the postoperative status: after 2 weeks, 4 weeks, 3 months, and 1 year. At each of these visits, a nasal endoscopy is performed to evaluate healing using the Ohio State University crusting scale from 0 to 3 (Table 3). Signs and symptoms of CSF leakage or fistula formation will be recorded. The patient will be asked to complete nasal symptom scoring (SNOT-22, SBI, VAS scoring) and EQ-5D-3L questionnaires to assess the quality of life. At FU3 and FU4, post-operative MR images will be evaluated as well.

**Table 3:** Ohio State University crusting scale

Crusting	
0	No crusting
1	Minimal crusting debrided with suction only
2	Moderate crusting (coating) requiring forceps debridement
3	Severe crusting (casting) causing obstruction

FU1 is an additional study visit, which is not part of the standard of care.

### Sample size {14}

Sample size calculation was performed according to the primary outcome, using an online power calculator for binary outcome non-inferiority trials (Sealed Envelope Ltd. Available from: https://www.sealedenvelope.com). This is a non-inferiority trial with a binary outcome using a one-sided alpha 0.05 and 80% power. The assumed success rate is 94.3% in the control arm, based on center-specific experience as well as the literature [14]. Data on the use of L-PRF for this indication is limited, but we assume a 95% success rate based on prior feasibility studies [8–10].

The statistical hypothesis for testing the treatment difference is presented as follows; H_0_: Δ ≤  − 0.07 tested against the alternative hypothesis H_A_: Δ >  − 0.07, where Δ is the difference between the success rates of experimental and control condition and − 0.07 is the non-inferiority difference. The non-inferiority limit was set at 0.07 considering (a) reported CSF leakage rates without commercially available fibrin sealant averaging 17.2% [15] and (b) important additional benefits of L-PRF compared to commercially available fibrin sealants including the completely autologous nature (eliminating immune reactions), presence of immunologic cells and reduced costs.

Based on this power calculation, 212 patients need to be included, 106 in each group. In order to account for potential missing follow-up information, failure of L-PRF preparation, or non-adherence to the allocation (expected drop-out rate less than 2%), 220 subjects will be enrolled, 110 in each group.

### Recruitment {15}

Patients will be recruited via the surgical planning tool of the electronic hospital record system. The planning tool is scanned weekly by the study coordinator and a list of probably eligible patients (all scheduled transsphenoidal surgeries) is presented to the surgeon.

This is a multicenter study, recruiting patients in three centers in Belgium. Considering the estimated enrollment rate of one out of two patients, we aimed to finish recruitment within 4 years, starting end of 2018. In UZ Leuven, the estimated number of yearly inclusions is 40. AZ Sint-Jan Brugge estimates to enroll 10–15 subjects per year, and in UZ Gent, 30–40 patients are expected to enroll yearly. However, recruitment for this study has been affected by the COVID-19 crisis in multiple ways. First, EETA is not considered as emergency surgery, so these procedures were postponed during the pandemic. Second, EETA is considered a high-risk procedure for disease transmission due to perioperative aerosol formation. Third, the involvement of the third center was postponed until 2022 because of regulatory delays related to the SARS-CoV-2 pandemic. Therefore, recruitment will be extended by an additional 2 years, aiming for the finalization of recruitment in 2024.

## Assignment of interventions: allocation

### Sequence generation {16a}

The patients are randomly divided in two groups, i.e., one arm to be treated with commercially available fibrin sealants and the other arm with L-PRF. The study coordinator has generated the randomization schedule, using an online randomization tool (https://www.sealedenvelope.com) for simple randomization with two treatment groups of equal size, no blocks, and no stratification factors.

### Concealment mechanism {16b}

The allocation list is uploaded into the Research Data Capturing solution (RedCap) system with access restricted to study personnel. Only one subject’s allocation can be released at a time, after confirming that the patient is suitable for randomization. The site-specific study coordinator or his/her delegate enrolls the patients.

### Implementation {16c}

Randomization is done during surgery using the Randomize-tool in RedCap by trained study personnel, i.e., the study coordinator. The arm to which the patient is allocated is communicated by telephone to the operation theatre staff.

## Assessment of interventions: blinding

### Who will be blinded? {17a}

The randomization is single-blinded, i.e., enrolled patients do not know which group they are allocated to. Blinding of surgical staff is not possible as L-PRF is clearly different from fibrin sealants. Bias of surgeons is reduced as much as possible by announcing the treatment arm only after surgical incision. Reporting bias during hospital follow-up is reduced as the paramedics at the neurosurgery hospitalization department do not have access to the randomization code. The ENT specialist performing postoperative endoscopic examinations and applying the crusting score is blinded. Researcher’s bias during follow-up visits is reduced by reblinding subject allocation after surgery. For analysis, group allocation will only be released after the statistical analysis has been performed.

### Procedure for unblinding if needed {17b}

Blinding may be lifted after completion of the last outpatient follow-up visit (FU4), or in case of any serious adverse event that is probably related to the study.

## Data collection and management

### Plans for assessment and collection of outcomes {18a}

Preoperative data is collected from the preoperative assessment of the anesthesiologist (medical history, comorbidity, and medication use) and neurosurgeon (surgical indication) available in the electronical patient files. Preoperative patient questionnaires are on paper source documents. Intraoperative data and used materials are collected from the surgical reports filed by the neurosurgeon and ENT. Data from the outpatient follow-up visits and postoperative imaging are available in the electronic files. Postoperative questionnaires are filled out on paper source documents. Data transfer from source documents to the RedCap data collection tool will be done by trained study personnel (research assistant). Regular data quality checks are performed in RedCap to ensure complete and accurate data transfer. Paper source documents will be stored for 10 years after completion of the study in a secure location at the study site.

### Plans to promote participant retention and complete follow-up {18b}

The postoperative follow-up visits are planned and notified to the patient before hospital discharge. If the patient is not present at one of these appointments, or if the clinical follow-up appointment is rescheduled outside the visit time window (+ / − 1 week for FU1 and FU2; + / − 2 weeks for FU3 and FU4), the patient is contacted by telephone to reschedule the visit. If this attempt is unsuccessful, the patient is considered lost to follow-up. In case of subject withdrawal, an exit note is added to the electronic Case Report Form (e-CRF) mentioning the withdrawal date and reason of withdrawal.

### Data management {19}

Data collection is performed partially on paper source documents (patient questionnaires) and partially on electronic source documents (patient medical records containing surgical and hospitalization reports, registration of used materials). Data are collected by the principal investigator or his designee and locally entered into the e-CRF (RedCap). RedCap is primarily a data collection tool that facilitates post study analysis based on qualitative data. Access to the e-CRF is strictly regulated and only possible after passing a test and with personal credentials. All operations on data are monitored and verified via a tracking system. Entries are verified by double entry (for example manual and automated summary of VAS, SNOT-22, and EQ-5D result), format checks e.g. integer, and warning messages if data are outside an expected range of values.

As appropriate, baseline characteristics will be reported by mean and standard deviation or number and proportions. The effect of the intervention on the primary outcome will be assessed by comparing the proportion of patients presenting with a postoperative CSF leakage within 3 months after surgery.

### Confidentiality {27}

Each patient is identified by a unique study subject number, to ensure the subject’s pseudonymity. All data are processed without identifiable reference to the patient. At each study site one secured identification list is available in the investigator site file. The list contains the code with the study subject number and the patient’s name, birth date, and hospital number.

### Plans for collection, laboratory evaluation, and storage of biological specimens for genetic or molecular analysis in this trial/future use {33}

As referred to in item 26b, no biological specimens will be collected.

## Statistical methods

### Statistical methods for primary and secondary outcomes {20a}

Data will be analyzed in GraphPad Prism software using a simple *t*-test, or nonparametric Mann–Whitney *U* test when not normally distributed, with Bonferroni Holm correction for multiple testing. A *p*-value ˂0.05 is considered statistically significant. The primary outcome, i.e., CSF leakage, will be analyzed in terms of a difference in risk between the two treatment groups. The mean difference between the two treatment groups will be reported as a 95% confidence interval. Secondary endpoints are calculated using simple *t*-test, or nonparametric Mann–Whitney *U* test when not normally distributed, or Fisher’s exact test or chi-squared test, based on the number of events.

### Interim analyses {21b}

Per protocol, no interim analyses will be performed.

### Methods for additional analyses (e.g., subgroup analyses) {20b}

Subset analyses will be performed for surgical variables such as recurrent surgery, tumor type (hormonal balance), size, and position (Wilson-Hardy classification, Knosp classification). Demographic variables including patient age, gender, body mass index, medication use, comorbidity, and smoking habits are also analyzed. The data will be statistically tested using a simple *t*-test or Mann–Whitney *U* test, or Fisher’s exact test or chi-squared test depending on normality, type of variable, and number of events. We do not intend to perform adjusted analyses.

### Methods in analysis to handle protocol non-adherence and any statistical methods to handle missing data {20c}

The results will be analyzed according to the following principles: the “intention-to-treat” (all randomized participants, irrespective of protocol adherence), the “per protocol” (only participants that were treated according to the protocol), and the “as treated” (all participants according to the treatment they received) principles. These three analyses will be compared to show how a lack of data possibly impacts the results. For the primary endpoint, no missing data are expected. For secondary endpoints (cost-effectiveness evaluation), the predictive mean of the other values within the arm will be used.

### Plans to give access to the full protocol, participant-level data, and statistical code {31c}

The study protocol has been registered and is available at ClinicalTtrials.gov (ID: NCT03910374). The statistical code and datasets analyzed during the current study are available from the corresponding author on reasonable request.

## Oversight and monitoring

### Composition of the coordinating center and trial steering committee {5d}

The principal investigator designed the trial protocol and CRF, while any revisions or changes are overseen by the principal investigator and study coordinator. The study coordinator manages the daily operation of the study and reports regularly to the principal investigator and sponsor. The study coordinator also submits annual progress and safety reports, which include adverse events. A supervisory committee, composed of experts in neurosurgery, otorhinolaryngology, and head and neck surgery assesses trial progress and patient safety annually. The study coordinator and principal investigator manage this committee, which is part of the trial management committee. The steering committee approves the final protocol and monitors the study progress, making changes as necessary for efficiency. There is no stakeholder and public involvement group for this study. The principal investigator and study coordinator will coordinate the publication of study reports.

### Composition of the data monitoring committee, its role and reporting structure {21a}

Due to the absence of high-risk populations (e.g., children or pregnant women) in the study and as the study does not involve any important additional medical risk, no medical steering committee monitoring was deemed necessary.

### Adverse events reporting and harms {22}

Harms and adverse events that may be expected in the study population include diabetes insipidus, anterior pituitary insufficiency, and nasal septum perforation. During hospitalization, harms will be systematically collected from documentation of clinical and radiological examinations, and daily nursing reports in the electronical patient files. At the outpatient follow-up visits (FU1-4), harms will be collected based on anamnesis and clinical examination. Additionally, three open-ended questions will be presented to the subjects:

- Did you suffer from any complaints?
- Have you been ill?
- Have you taken any medication?

During the study, all adverse events are recorded and notified to the sponsor in an annual progress report. In agreement with the local law, serious adverse events are reported immediately, after first knowledge, to the sponsor and ethical committee, accompanied by detailed written reports. A serious adverse event is defined as any untoward medical occurrence or effect that results in death, is life-threatening, requires hospitalization or prolongation of existing hospitalization, results in persistent or significant disability or incapacity, or is a congenital anomaly or birth defect. Such serious adverse events will be reported in the final manuscript of the study. The sponsor shall keep detailed reports of all adverse events which are reported. For reported death of a subject, the investigator shall supply the institutional Ethical Committee with any additional information requested.

### Frequency and plans for auditing trial conduct {23}

UZ/KU Leuven applies a risk-based approach to monitoring clinical research studies sponsored by them, which involves reviewing the studies upon registration, assessing their level of risk, and determining the monitoring strategy accordingly. Monitoring may also be requested by the UZ/KU Leuven leadership team, Ethics Committee, Competent Authorities, or the study’s financiers, regardless of the risk assessment outcome.

### Plans for communicating important protocol amendments to relevant parties (e.g., trial participants, ethical committees) {25}

Protocol amendments shall be filed to the institution’s Ethical Committee for approval, after agreement of the principal investigator. The trial register will be updated. If the amendment might affect the safety or procedure of already enrolled subjects, they will be notified and asked to sign an additional updated informed consent form.

## Dissemination plans {31a}

Upon finalization of the study, the institutional Ethical Committee will be notified. The results of the study will be submitted to a peer-reviewed scientific journal and presented at international conferences. The results will be disseminated regardless of the magnitude or the direction of the effect. If requested by the patient, he/she will be updated about the results via e-mail when available.

Substantial contributions to the design, conduct, interpretation, and reporting of the clinical trial will be recognized through the granting of authorship on the final trial report. We do not intend to employ professional writers.

## Discussion

The design and rationale of a multicenter, prospective, randomized controlled trial studying the role of L-PRF in EETA is discussed. The primary aim of the study is to investigate non-inferiority of L-PRF compared to commercially available fibrin sealants, the current standard of care. Additionally, we will assess the safety and cost-effectiveness of L-PRF for dural closure in EETA, identify possible risk factors for reconstruction failure, and investigate the influence on post operative rhinological symptoms and interference with postoperative imaging. The results of this trial will provide further insight in the efficiency of dural closure. As CSF leakage is an important complication of EETA, this study is of major importance to the field of neurosurgery, skull base surgery, and ENT surgery. Some preliminary pilot studies reported the feasibility of L-PRF as a closure aid in neurosurgery [8–10, 16]. In order to explore further possibilities of the use of L-PRF in cranial surgery, we are currently performing a similar randomized controlled trial in which L-PRF is compared to commercially available fibrin sealants for dural closure in supra- and infratentorial surgeries [17].

L-PRF has been reported to enhance wound healing by providing growth factors to the surrounding tissue [11, 12]. This study represents the first large-scale randomized controlled trial comparing L-PRF to the current standard of care. With the intended sample size, we aim to show non-inferiority of L-PRF with respect to CSF leakage. The results of this trial can impact future decision-making for skull base reconstruction in EETA.

## Trial status

Recruitment of this clinical trial started on 7 November 2018. We are currently recruiting under protocol version 5 (9 November 2021). Study completion is estimated by February 2025.

## Supplementary Information

**Additional file 1: Supplementary table S1.** World Health Organization Trial Registration Data Set {2b}.

**Additional file 2. **Informed consent.

**Additional file 3. **SPIRIT 2013 Checklist.

## Data Availability

The final dataset and study reports will be included in the Trial Master File for the study and archived at the sponsor and participating sites for 20 years after study finalization. The files will be accessible for dedicated personnel at the study site. The datasets used and/or analyzed during the current study are available from the corresponding author on reasonable request.

## Abbreviations

CSF: Cerebrospinal fluid
e-CRF: Electronic Case Report Form
EETA: Endoscopic endonasal transsphenoidal approach
EMA: European Medicines Agency
EQ-5D: EuroQol five Dimensions questionnaire
FDA: Food and Drug Administration
FU: Follow-up
L-PRF: Leukocyte- and platelet-rich fibrin
RedCap: Research Data Capturing solution
SBI: Skull Base Inventory
SNOT-22: Sinonasal outcome test
QALY: Quality-adjusted life-year
QoL: Quality of life
VAS: Visual analog scale
