# Supplementary material for: Leukocyte- and platelet-rich fibrin in endoscopic endonasal skull base reconstruction: study protocol for a multicenter prospective, parallel-group, single-blinded randomized controlled non-inferiority trial
Source: Trials. 2023 Jul 31;24:488. doi: 10.1186/s13063-023-07492-w (PMC10388446; doi:10.1186/s13063-023-07492-w)
Supplement: Supplementary file 1 — Additional file 1: Supplementary table S1. World Health Organization Trial Registration Data Set {2b}. [file 13063_2023_7492_MOESM1_ESM.docx]

**Supplementary table S1: World Health Organization Trial Registration Data Set** **{2b}**

| Item |  |
| --- | --- |
| 1. Primary registry and trial identifying number | Clinicaltrials.gov NCT03910374 |
| 2. Date of registration in primary registry | 10 April 2019 |
| 3. Secondary identifying numbers | Ethics Committee KU/UZ Leuven S61636  FWO T003018N |
| 4. Sources of monetary or material support | FWO TBM grant T003018N |
| 5. Primary sponsor | UZ Leuven |
| 6. Secondary sponsor | NA |
| 7. Contact for public queries | Birgit Coucke, [Birgit.coucke@kuleuven.be](mailto:Birgit.coucke@kuleuven.be), +32 16 34 55 70  Herestraat 49 Box 811 3000 Leuven |
| 8. Contact for scientific queries | Prof. Dr. Tom Theys, [Tom.theys@uzleuven.be](mailto:Tom.theys@uzleuven.be), +32 16 34 42 90  Department of Neurosurgery, University Hospitals Leuven, Leuven, Belgium  Herestraat 49 Box 7003 3000 Leuven |
| 9. Public title | Application of Leukocyte- and Platelet-Rich Fibrin (L-PRF) in Neurosurgery and Otorhinolaryngology |
| 10. Scientific title | Leukocyte- and Platelet-Rich Fibrin (L-PRF) in endoscopic endonasal skull base reconstruction: a multicenter prospective, parallel-group, single-blinded randomized controlled non-inferiority trial. |
| 11. Countries of recruitment | Belgium |
| 12. Health condition(s) or problem(s) studied | Skull base reconstruction |
| 13. Intervention(s) | Experimental: Leukocyte- and Platelet-Rich Fibrin (L-PRF), blood-derived autologous material  Control: commercially available fibrin sealants |
| 14. Key inclusion and exclusion criteria | Inclusion: Patients with lesions in the sellar or parasellar region undergoing transsphenoidal surgery; informed consent  Exclusion: underlying rhinological condition that may interfere with the obtained results, participation to other clinical trials with study drugs or devices  Eligible ages: 18 years and older  Eligible sexes: all  Accepts healthy volunteers: no |
| 15. Study type | Randomized, parallel, single-blinded (patient) interventional multicentric trial. Randomization sequence is generated via [www.sealedenvelope.com](http://www.sealedenvelope.com) by the study coordinator and concealed using RedCap. Primary purpose: prevention |
| 16. Date of first enrollment | November 7^th^, 2018 |
| 17. Target sample size | 220 |
| 18. Recruitment status | Recruiting |
| 19. Primary outcome | Incidence of cerebrospinal fluid leakage/rhinorrhea at 12 weeks postoperative |
| 20. Key secondary outcome(s) | Cost-effectiveness of L-PRF (time frame: 1 year postoperative)  Identification of risk factors (time frame: 1 year postoperative)  Assessment of potential interference of reconstruction material with post-operative imaging (time frame: 1 year postoperative)  Analysis of the effect of the treatment on QoL and post-operative rhinological symptoms (time frame: 1 year postoperative) |
