# Supplementary material for: Leukocyte- and platelet-rich fibrin in endoscopic endonasal skull base reconstruction: study protocol for a multicenter prospective, parallel-group, single-blinded randomized controlled non-inferiority trial
Source: Trials. 2023 Jul 31;24:488. doi: 10.1186/s13063-023-07492-w (PMC10388446; doi:10.1186/s13063-023-07492-w)
Supplement: Supplementary file 2 — Additional file 2. Informed consent. [file 13063_2023_7492_MOESM2_ESM.docx]

**Informed consent for the patient**

Full title of the trial:

Role of Leukocyte- and Platelet-Rich Fibrin membranes in endoscopic endonasal skull base reconstruction.

1. **Introduction**

With this information, we would like to inform you about this trial, and we kindly ask your cooperation. Please take your time to read the information carefully and to discuss this information with others. One of the members of the research team will discuss the information in this document and is available to answer possible questions. Please read these few pages of information carefully and ask any questions you want to the investigator or his/her representative.

1. **What is the purpose of this trial?**

Recent developments in surgical techniques to access the skull base and brain via the nose and through the sinuses, have led to the need of closure of this access route. At the moment, the defect that is created through this pathway is restored with foreign material and closed with a commercial glue (Tachosil® and Tisseel®).

With this trial we want to investigate the efficacy of an autologous fibrin membrane of leukocytes and platelets (L-PRF) and want to compare this with the standard procedure in which synthetic glues/materials are used. We also want to make an evaluation of the costs and mechanisms of both techniques. Furthermore, we are interested in possible complaints that can exist after the surgery.

This autologous L-PRF will be made after a simple processing of your blood (maximum 10 tubes) during the surgery. This material knew his first application in dental practices.

Our goal is to reach 220 patients in the study, which will last 3 years.

1. **Why am I chosen to participate?**

We invite you to participate in this trial because you will undergo skull base surgery with access through the nose and the sinuses.

The brain and spinal cord are surround by a protective cerebrospinal fluid. This fluid is surrounded by a meninx (dura). The surgeon needs to make sure that the defect in this dura and the access to the skull base are well closed to avoid leakage of the cerebrospinal fluid.

Different techniques such as the commercial/synthetic standard products (Tachosil® and Tisseel®) and the autologous L-PRF are already used in this hospital.

In this trial, we want to investigate the efficacy and cost of L-PRF compared to the standard techniques together with a registration of possible complications and symptoms.

1. **Should I participate?**

Participation in the study is completely voluntary.  You may decide independently to participate or not.  If you decide to participate, you will be asked to sign a consent form.  If you decide to participate, you have the freedom to stop your participation at any time without giving any reason.  If you decide not to participate, this will have no impact on the medical care you receive.

1. **What will happen if I participate?**

When you decide to participate, we will ask you to sign the informed consent attached to this document.

Participation in the trial will start from the moment you sign until the last check up (about 1 year after the surgery). This will include 5 consultations in the hospital:

1 previous to the surgery (screening/today), the surgery, 4 follow-up appointments after 2, 4, 12 and 52 weeks after the surgery.

The follow up visit at 2 and 52 weeks after surgery are extra moments needed in this trial. All other assessments will be performed on time points where you will already be expected in the hospital.

At the moment it is not known which technique is superior the close the defect in the skull base. Therefore we want to compare the commercial standard products with the autologous L-PRF.

Participants in the trial will be divided in 2 groups: 1 group will be treated with L-PRF and 1 group will be treated with the standard products. This is necessary to compare both techniques. This division is random and done by chance. Your doctor knows which treatment you received; you will only be informed at the end of the trial.

Both techniques are already extensively used in this hospital with reassuring results, although they were never compared.

What will be expect at every consultation?

**-1 Screening**: Medical history will be asked; a questionnaire will be filled out concerning the quality of life and possible nasal symptoms. An evaluation of the MRI images will be performed.

**0 Randomization**: endoscopic (with a camera) surgery of the brain through the nose and sinuses.

50% of the patients will be treated with L-PRF and 1 group will be treated with the standard products (Tachosil® and Tisseel®).

**1. Follow up 1**: this visit will occur 2 weeks after the surgery, a nasal endoscopy (little camera accessing the nose) will be performed to evaluation the healing. Questionnaires concerning quality of life and symptoms will be filled out.

**2. Follow up 2**: this visit will occur 4 weeks after the surgery and the same parameters as in follow up visit 1 will be examined.

**3. Follow up 3**: this visit will occur 12 weeks after the surgery and the same parameters as in follow up visit 1 will be examined, with an MRI scan.

**4. Follow up 4**: this visit will occur 1 year after the surgery. The same parameters as in follow up 3 will be examined.

1. **Insurance**

In accordance with the Belgian Law of May 7, 2004 concerning experiments on the human person, even if the client is not at fault, they are liable for all damages incurred by the participant and/or his assignees and which is directly or indirectly related to the investigation.  The UZ Leuven has taken out insurance to cover this liability.  lf you were to incur damage resulting from your participation in this study, damage will therefore be compensated in accordance with the Belgian Law of May 7, 2004.  In case of any problems with the study, you are therefore requested to contact us as soon as possible.

1. **Are there advantages or risks associated with participation?**

Whether you choose to participate has no impact on the medical care you receive.  This is a study in which some information is recorded.  The treatment is not affected by your participation in the study.  Therefore, participation in the study can provide no benefit, no risk and no additional cost.

1. **What are the possible risks of participation?**

You will undergo a planned surgery. Possible risks and discomforts that can be expected are the usual risks and discomforts that can accompany the surgery.

There are no side-effects expected. The L-PRF is manufactured from your blood, this means that there is little chance of rejecting.

1. **What are the benefits of participating?**

If you agree to take part in this study, the L-PRF may or may not prove beneficial in comparison with the standard products in treating the defect in the skull base. The information obtained thanks to this study may contribute to a better knowledge of the use of these products in future patients.

We expect a possible faster recovery in one of the two techniques.

1. **Will my participation be treated confidential?**

Yes. Your participation in the study means that you agree to the investigator collecting data about you and to the study sponsor using these data for research purposes and in connection with scientific and medical publications.

You are entitled to ask the investigator what data are being collected about you and what is their use in connection with the study. This data concerns your current clinical situation but also some of your background, the results of examinations carried out within the context of care of your health in accordance with the current standards and obviously the results of examinations required by the protocol.

The investigator has a duty of confidentiality with the data collected.

This means that he/she undertakes not only never to reveal your name in the context of a publication or conference but also that he/she will encode (your identity will be replaced by an ID code in the study).

The investigator and his/her team will therefore be the only ones to be able to establish a link between the data transmitted throughout the study and your medical records.

These rights are guaranteed by the Law of 8 December 1992 on the protection of privacy in relation to the processing of personal data and by the Law of 22 August 2002 on patient rights.

1. **What will happen with the results of the study?**

The results of this study will be published but your identity remains anonymous. It is possible to receive a copy of these results from the research team.

1. **Approval of this Investigation**

This project has received a positive opinion for the Committee on Medical Ethics of the University Hospital/University of Leuven.

After reading this information, you will have plenty of time to think and decide whether to participate in this study.  If you give permission to participate, you must sign the appropriate consent form.

1. **Contact details:**

If you need further information, but also if you have problems or concerns, you can contact the investigator Prof. Dr. Laura Van Gerven or a member of his/her research team on the following telephone number (016/33.23.42).

Outside consulting hours, contact the A&E department of your hospital, indicating that you are taking part in a clinical study. Your records will contain information of use to the on-call doctor in relation to this clinical study.

If you have any questions relating to your rights as a participant in a clinical study, you can contact the patient rights ombudsman of your institution on this telephone number: telephone details. If necessary, he/she can put you in contact with the ethics committee.

**Thank you for taking the time to read this information.**

**Informed consent (for patient)**

**The role of Leukocyte- and Platelet-Rich Fibrin membranes**  
**in endoscopic endonasal skull base reconstruction**

- I declare that I have been informed of the nature of the study, its purpose, its duration, any risks and benefits and what is expected of me. I have taken note of the information document version 2 (30/8/2018) and the appendices to this document.
- I have had sufficient time to think about it and discuss it with a person of my choice, such as my GP or a member of my family.
- I have had the opportunity to ask any questions that came to mind and have obtained a satisfactory response to my questions.
- I understand that my participation in this study is voluntary and that I am free to end my participation in this study without this affecting my relationship with the therapeutic team in charge of my health.
- I understand that data about me will be collected throughout my participation in this study and that the investigator and the sponsor of the study will guarantee the confidentiality of these data.
- I have received a copy of the information to the participant and the informed consent form.

**I agree with participation in this trial and have given consent before any study procedures were carried out.**

Patient:

______________________ ___________________ __________________

Name Signature Date

Legal representative if the patient is not able to consent him/herself:

______________________ ___________________ __________________

Name Signature Date

Researcher:

I, the undersigned investigator/clinical study assistant, confirm that I have verbally provided the necessary information about the study and have given the participant a copy of the information document.

______________________ ___________________ __________________

Name Signature Date

**Informed consent (for researcher)**

**The role of Leukocyte- and Platelet-Rich Fibrin membranes**  
**in endoscopic endonasal skull base reconstruction**

- I declare that I have been informed of the nature of the study, its purpose, its duration, any risks and benefits and what is expected of me. I have taken note of the information document version 2 (30-8-2018) and the appendices to this document.
- I have had sufficient time to think about it and discuss it with a person of my choice, such as my GP or a member of my family.
- I have had the opportunity to ask any questions that came to mind and have obtained a satisfactory response to my questions.
- I understand that my participation in this study is voluntary and that I am free to end my participation in this study without this affecting my relationship with the therapeutic team in charge of my health.
- I understand that data about me will be collected throughout my participation in this study and that the investigator and the sponsor of the study will guarantee the confidentiality of these data.
- I have received a copy of the information to the participant and the informed consent form.

**I agree with participation in this trial and have given consent before any study procedures were carried out.**

Patient:

______________________ ___________________ __________________

Name Signature Date

Legal representative if the patient is not able to consent him/herself:

______________________ ___________________ __________________

Name Signature Date

Researcher:

I, the undersigned investigator/clinical study assistant, confirm that I have verbally provided the necessary information about the study and have given the participant a copy of the information document.

______________________ ___________________ __________________

Name Signature Date
